# Emerging vaccine-breakthrough SARS-CoV-2 variants

**Published:** 2021-09-09

**Authors:** Rui Wang, Jiahui Chen, Yuta Hozumi, Changchuan Yin, Guo-Wei Wei

**Affiliations:** 1 Department of Mathematics, Michigan State University, MI 48824, USA.; 2 Department of Mathematics, Statistics, and Computer Science, University of Illinois at Chicago, Chicago, IL 60607, USA; 3 Department of Electrical and Computer Engineering, Michigan State University, MI 48824, USA.; 4 Department of Biochemistry and Molecular Biology, Michigan State University, MI 48824, USA.

**Keywords:** COVID-19, SARS-CoV-2, receptor-binding domain, co-mutations, variants, vaccine-breakthrough, vaccine-escape, vaccine-resistant, infectivity

## Abstract

The recent global surge in coronavirus disease 2019 (COVID-19) infections have been fueled by new severe acute respiratory syndrome coronavirus 2 (SARS-CoV-2) variants, namely Alpha, Beta, Gamma, Delta, etc. The molecular mechanism underlying such surge is elusive due to the existence of 28,554, including 4,653 non-degenerate mutations on the spike (S) protein, which is the target of most COVID-19 vaccines. The understanding of the molecular mechanism of SARS-CoV-2 transmission and evolution is a prerequisite to foresee the global trend of emerging vaccine-breakthrough SARS-CoV-2 variants and the design of mutation-proof vaccines and monoclonal antibodies (mAbs). We integrate the genotyping of 1,489,884 SARS-CoV-2 genomes isolated from patients, a library collection of 130 human antibodies, tens of thousands of mutational data points, topological data analysis (TDA), and deep learning to reveal SARS-CoV-2 evolution mechanism and forecast emerging vaccine-escape variants. We show that infectivity-strengthening and antibody-disruptive co-mutations on the S protein receptor-binding domain (RBD) can quantitatively explain the infectivity and virulence of all prevailing variants. We demonstrate that Lambda is as infectious as Delta but is more vaccine-resistant. We analyze emerging vaccine-breakthrough co-mutations in 20 COVID-19 devastated countries, including the United Kingdom (UK), the United States (US), Denmark (DK), Brazil (BR), Germany (DE), Netherlands (NL), Sweden (SE), Italy (IT), Canada (CA), France (FR), India (IN), and Belgium (BE), etc. We envision that natural selection through infectivity will continue to be a main mechanism for viral evolution among unvaccinated populations, while antibody disruptive co-mutations will fuel the future growth of vaccine-breakthrough variants among fully vaccinated populations. Finally, we have identified the following sets of co-mutations that have the great likelihood of becoming dominant: [A411S, L452R, T478K], [L452R, T478K, N501Y], [V401L, L452R, T478K], [K417N, L452R, T478K], [L452R, T478K, E484K, N501Y], and [P384L, K417N, E484K, N501Y]. We predict they, particularly the last four, will break through existing vaccines. We foresee an urgent need to develop new vaccines that target these co-mutations.

## 1 Introduction

The death toll of coronavirus disease 2019 (COVID-19) caused by severe acute respiratory syndrome coronavirus 2 (SARS-CoV-2) has exceeded 4.4 million in August 2021. Tremendous efforts in combating SARS-CoV-2 have led to several authorized vaccines, which mainly target the viral spike (S) proteins. However, the emergence of mutations on the S gene has resulted in more infectious variants and vaccine breakthrough infections. Emerging vaccine breakthrough SARS-CoV-2 variants pose a grand challenge to the long-term control and prevention of the COVID-19 pandemic. Therefore, forecasting emerging breakthrough SARS-CoV-2 variants is of paramount importance for the design of new mutation-proof vaccines and monoclonal antibodies (mABs).

To predict emerging breakthrough SARS-CoV-2 variants, one must understand the molecular mechanism of viral transmission and evolution, which is one of the greatest challenges of our time. SARS-CoV-2 entry of a host cell depends on the binding between S protein and the host angiotensin-converting enzyme 2 (ACE2), primed by host transmembrane protease, serine 2 (TMPRSS2) [1]. Such a process inaugurates the host’s adaptive immune response, and consequently, antibodies are generated to combat the invading virus either through direct neutralization or non-neutralizing binding [2, 3]. S protein receptor-binding domain (RBD) is a short immunogenic fragment that facilitates the S protein binding with ACE2. Epidemiological and biochemical studies have suggested that the binding free energy (BFE) between the S RBD and the ACE2 is proportional to the infectivity [1,4–7]. Additionally, the strong binding between the RBD and mAbs leads to effective direct neutralization [8–10]. Therefore, RBD mutations have dominating impacts on viral infectivity, mAb efficacy, and vaccine protection rates. Mutations may occur for various reasons, including random genetic drift, replication error, polymerase error, host immune responses, gene editing, and recombinations [11–15]. Being beneficial from the genetic proofreading mechanism regulated by NSP12 (a.k.a RNA-dependent RNA polymerase) and NSP14 [16, 17], SARS-CoV-2 has a higher fidelity in its replication process than the other RNA viruses such as influenza. Nonetheless, near 700 non-degenerate mutations are observed on RBD, contributing many key mutations in emerging variants, i.e., N501Y for Alpha, K417N, E484K, and N501Y for Beta, K417T, E484K, and N501Y for Gamma, L452R and T478K for Delta, L452Q and F490S for Lambda, etc [18]. Given the importance of the RBD for SARS-CoV-2 infectivity, vaccine efficacy, and mAb effectiveness, it is imperative to understand the mechanism governing RBD mutations.

In June 2020, when there were only 89 non-degenerated mutations on the RBD, and the highest observed mutational frequency was only around 50 globally, we were able to show that natural selection underpins SARS-CoV-2 evolution, based on the genotyping of 24,715 SARS-CoV-2 sequences isolated patients and a topology-based deep learning model for RBD-ACE2 binding analysis [19]. In the same work, we predicted that RBD residues 452 and 501 “have high chances to mutate into significantly more infectious COVID-19 strains” [19]. Currently, these residues are the key mutational sites of all prevailing SARS-CoV-2 variants. We further foresaw a list of 1,149 most likely RBD mutations among 3686 possible RBD mutations [19]. Up to date, every one of the observed 683 RBD mutations belongs to the list. In April 2021, we demonstrated that all the 100 most observed RBD mutations of 651 existing RBD mutations from 506,768 viral genomes had enhanced the binding between RBD and ACE2, resulting in more infectious variants [18]. The odd for these 100 most observed mutations to be there accidentally is smaller than one chance in 1.2 nonillions (2^100^ ≈ 1.2 × 10^30^)^1^. There is no double that natural selection via viral infectivity, rather than any other competing theories [11–15], is the dominating mechanism for SARS-CoV-2 transmission and evolution. This mechanistic discovery lays the foundation for forecasting future emerging SASR-CoV-2 variants.

Understanding SARS-CoV-2 variant threats to current vaccines and mAbs is another urgent issue facing the scientific community [20]. The World Health Organization (WHO) identified variants of concern (VOCs) and variants of interest (VOIs). The former describes variants that have an increment in the transmissibility and virulence, or adversely affect the effectiveness of vaccines, therapeutics, and diagnostics with clear clinical correlation evidence. The latter describes variants that carry genetic changes, which are predicted or known to reduce neutralization by antibodies generated against vaccination, the efficacy of treatments, and affect transmissibility, virulence, disease severity, immune escape, diagnostics, etc., which cause significant community transmission and suggest an emerging risk to the public. Currently, WHO listed four VOCs, i.e., variants B.1.1.7 (Alpha) [21–23], B.1.351 (Beta) [22,24], P.1 (Gamma) [22], and B.1.617.2 (Delta) [25]), and four VOIs, i.e., variants B.1.525 (Eta) [26], B.1.526 (Iota) [26, 27], B.1.617.1 (Kappa) [28], C.37 (Lambda) [29], and B.1.621 (Mu) (A general introduction about the prevailing and emerging variants is given in Section S1 of the Supporting Information.). Our hypothesis is that the severity of variants to infectivity, vaccine efficacy, and mAbs effectiveness depends mainly on how the associated RBD mutations impact the binding with ACE2 and antibodies. Based on this hypothesis, we collected and analyzed a library of antibodies and unveiled that most of the RBD mutations would weaken the binding of S protein and antibodies and disrupt the efficacy and reliability of antibody therapies and vaccines [20]. We predicted “the urgent need to develop new mutation-resistant vaccines and antibodies and prepare for seasonal vaccination” in early 2021 [20]. We further identified vaccine-escape (i.e., vaccine-breakthrough) mutations and fast-growing mutations [18]. Our predictions of the threats from VOCs and VOIs were in great agreement with experimental data [30].

The objective of this work is to forecast emerging SARS-CoV-2 variants that pose an imminent threat to combating COVID-19 and long-term public health. To this end, we carry out an RBD-specific analysis of SARS-CoV-2 co-mutations involving a wide variety of combinations of 683 unique single mutations on the RBD. We take a unique approach that integrates viral genotyping of 1,489,884 complete genome sequences isolated from patients, algebraic topology algorithms that won the worldwide competition in computer-aided drug discovery [31], deep learning models trained with tens of thousands of mutational data points [20,30], and a library of 130 SARS-CoV-2 antibody structures. By analyzing the frequency, binding free energy (BFE) changes, and antibody disruption counts of RBD co-mutations, we reveal that nine RBD co-mutation sets, namely [L452R, T478K], [L452Q, F490S], [E484K, N501Y], [F490S, N501Y], [S494P, N501Y], [K417T, E484K, N501Y], [K417N, L452R, T478K], [K417N, E484K, N501Y], and [P384L, K417N, E484K, N501Y], may strongly disrupt existing vaccines and mAbs with relatively high infectivity and transmissibility among the populations. We predict that low-frequency co-mutation sets [A411S, L452R, T478K], [L452R, T478K, N501Y], [V401L, L452R, T478K], and [L452R, T478K, E484K, N501Y] are on the path to become dangerous new variants. The associated new mutations, P384L, V401L, and A411S, call for the new design of boosting vaccines and mAbs.

## 2 Results

### 2.1 Vaccine-breakthrough S protein RBD mutations

**Figure 1:**
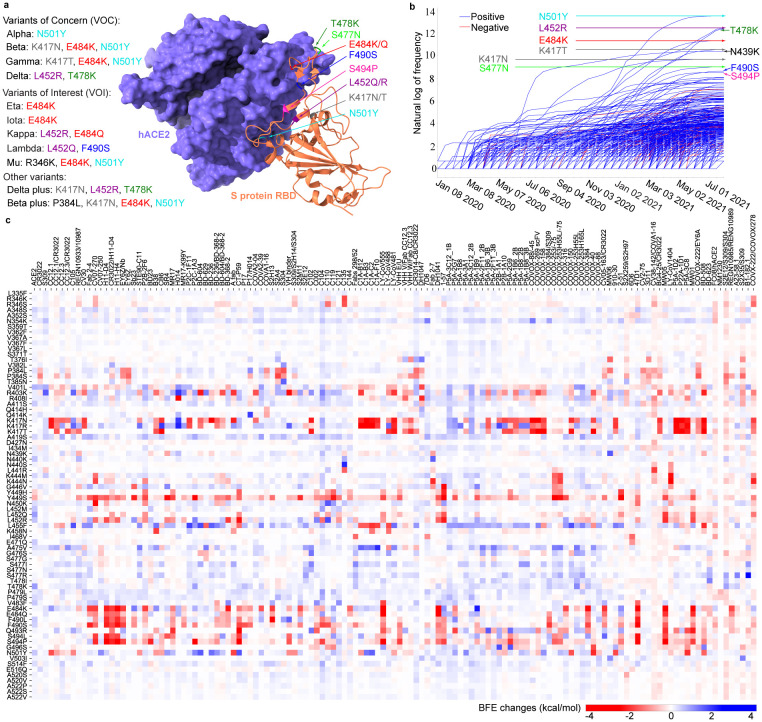
Most significant RBD mutations. **a** The 3D structure of SARS-CoV-2 S protein RBD and ACE2 complex (PDB ID: 6M0J). The RBD mutations in ten variants are marked with color. **b** Illustration of the time evolution of 455 ACE2 binding-strengthening RBD mutations (blue) and 228 ACE2 binding-weakening RBD mutations (red). The *x*-axis represents the date and the *y*-axis represents the natural log of frequency. There has been a surge in the number of infections since early 2021. **c** BFE changes of RBD complexes with ACE2 and 130 antibodies induced by 75 significant RBD mutations. A positive BFE change (blue) means the mutation strengthens the binding, while a negative BFE change (red) means the mutation weakens the binding. Most mutations, except for vaccine-resistant Y449H and Y449S, strengthen the RBD binding with ACE2. Y449S and K417N are highly disruptive to antibodies.

To understand the molecular mechanisms of vaccine-escape mutations, we analyze single nucleotide polymorphisms (SNPs) of 1,489,884 complete SARS-CoV-2 genome sequences, resulting in 683 non-degenerate RBD mutations and their associated frequencies. A full set of mutation information is available on our interactive web page Mutation Tracker. The infectivity of each mutation is mainly determined by the mutation-induced BFE change to the binding complex of RBD and ACE2. To estimate the impact of each mutation on vaccines, we collect a library of 130 antibody structures (Supporting Information S2.1.2), including Food and Drug Administration (FDA)-approved mAbs from Eli Lilly and Regeneron. For a given RBD mutation, its number of antibody disruptions is given by the number of antibodies whose mutation-induced antibody-RBD BFE changes are smaller than −0.3kcal/mol (A list of names for antibodies that are disrupted by mutations can be found in the Supporting Information S2.1.1.). BFE changes following mutations are predicted by our deep learning model, TopNetTree [32]. We have created an interactive web page, Mutation Analyzer, to list all RBD mutations, their observed frequencies, their RBD-ACE2 BFE changes following mutations, their number of antibody disruptions, and various ranks. Figure 1 illustrates RBD mutations associated with prevailing SARS-CoV-2 variants, time evolution trajectories of all RBD mutations, and the BFE changes of RBD-ACE2 and 130 RBD-antibodies induced by 75 significant mutations. A summary of our analysis is given in Table 1.

First, the 10 most observed or fast-growing RBD mutations are N501Y, L452R, T478K, E484K, K417T, S477N, N439K, K417N, F490S, and S494P, as shown in Table 1. Inclusively, these top mutations strengthen their BFEs and become more infectious, following the natural selection mechanism [19]. Figure 1b shows that the frequencies of the top three mutations increased dramatically since 2021 due to Alpha, Beta, Gamma, Delta, and other variants. Second, among the top 25 most observed RBD mutations, T478K, L452Q N440K, L452R, N501Y, N501T, F490S, A475V, and P384L are the 8 most infectious ones judged by their ability to strengthen the binding with ACE2, as shown in Figure 1c. The BFE changes of S protein and ACE2 for mutation T478K is nearly 1.00 kcal/mol, which strongly enhances the binding of the RBD–ACE2 complex [33]. Together with L452R (BFE change: 0.58kcal/mol), T478K makes Delta the most infectious variant in VOCs. Third, among the top 25 most observed RBD mutations, Y449S, S494P, K417N, F490S, L452R, E484K, K417T, E484Q, L452Q, and N501Y are the 10 most antibody disruptive ones, judged by their interactions with 130 antibodies shown in Figure 1c. It can be seen that mutations L452R, E484K, K417T, K417N, F490S, and S494P disrupt more than 30% of antibody-RBD complexes, while mutations E484K and K417T may disrupt nearly 30% antibody-RBD complexes, indicating their disruptive ability to the efficacy and reliability of antibody therapies and vaccines. The most dangerous mutations are the ones that are both infectivity-strengthening and antibody disruptive. Four RBD mutations, N501Y, L452R, F490S, and L452Q, appear in both lists and are key mutations in WHO’s VOC and VOI lists. Among them, F490S and L452Q are the key RBD mutations in Lambda, making Lambda a more dangerous emerging variant than Delta. Note that high-frequency mutation S477N does not significantly weaken any antibody and RBD binding, and thus does not appear in any prevailing variants.

### 2.2 Vaccine-breakthrough S protein RBD co-mutations

**Figure 2:**
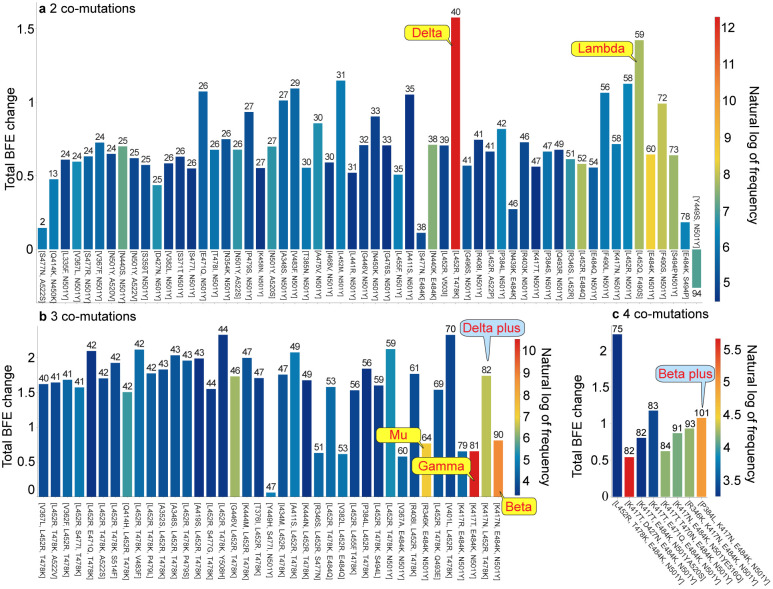
Properties of RBD co-mutations. **a** Illustration of RBD 2 co-mutations with a frequency greater than 90. **b** Illustration of RBD 3 co-mutations with a frequency greater than 30. **c** Illustration of RBD 2 co-mutations with a frequency greater than 20. Here, the *x*-axis lists RBD co-mutations and the *y*-axis represents the predicted total BFE change of each set of RBD co-mutations. The number on the top of each bar is the AI-predicted number of antibody and RBD complexes that may be significantly disrupted by the set of RBD co-mutations, and the color of each bar represents the natural log of frequency for each set of RBD co-mutations. (Please check the interactive HTML files in the Supporting Information S2.2.4 for a better view of these plots.)

The recent surge in COVID-19 infections is due to the occurrence of RBD co-mutations that combine two or more infectivity-strengthening mutations. The most dangerous future SARS-CoV-2 variants must be RBD co-mutations that combine infectivity-strengthening mutation(s) with antibody disruptive mutation(s). A list of 1,139,244 RBD co-mutations that are decoded from 1,489,884 complete SARS-CoV-2 genome sequences can be found in Section S2.1.3 of the Supporting Information, and all of the non-degenerate RBD co-mutations with their frequencies, antibody disruption counts, total BFE changes, and the first detection dates and countries can be found in Section S2.1.4 of the Supporting Information. Figure 2 illustrates the properties of S protein RBD 2, 3, and 4 co-mutations. The height of each bar shows the predicted total BFE change of each set of co-mutations on RBD, the color represents the natural log of frequency for each set of RBD co-mutations, and the number at the top of each bar is the AI-predicted number of antibody-RBD complexes that each set of RBD co-mutations may disrupt based on a total of 130 RBD and antibody complexes. Notably, for a specific set of co-mutations, the higher the number at the top of the bar is, the stronger ability to break through vaccines will be. From Figure 2, RBD 2 co-mutation set [L452R, T478K] (Delta variant) has the highest frequency (219,362) and the highest BFE change (1.575 kcal/mol). Moreover, the Delta variant would disrupt 40 antibody-RBD complexes, suggesting that Delta would not only enhance the infectivity but also be a vaccine breakthrough variant. Moreover, [L452Q, F490S] (Lambda) is another co-mutation with high frequency, high BFE changes (1.421 kcal/mol), and high antibody disruption count (59). In addition, Lambda is considered to be more dangerous than Delta due to its higher antibody disruption count. Further, [R346K, E484K, N501Y] (Mu variant) has a BFE change of 0.768 kcal/mol and high antibody disruption count (60). It is not as infectious as Delta and Lambda, but has a similar ability as Lambda in escaping vaccines. Note that among all VOCs and VOIs, Beta has the highest ability to break through vaccines, but its infectivity is relatively low (BFE change: 0.656 kcal/mol). Furthermore, high-frequency 2 co-mutation sets [E484K, N501Y], [F490S, N501Y], and [S494P, N501Y] are all considered to be the emerging variants that have the potential to escape vaccines. From Figure 2, three 3 co-mutation sets [R345K, E484K, N501Y] (Mu), [K417T, E484K, N501Y] (Gamma), and [K417N, E484K, N501Y] (Beta) draw our attention. They are all the prevailing three co-mutations with moderate BFE changes but very high antibody disruption count (more than 60). With a BFE change of 1.4 kcal/mol and antibody disruption count of 82, co-mutation set [K417N, L452R, T478K] (Delta plus) appears to be more dangerous than all of the current VOCs and VOIs. For 4 co-mutations in Figure 2 c, [P384L, K417N, E484K, N501Y] (Beta plus) could penetrate all vaccines due to its highest antibody disruption count of 101. We would like to address that all of the co-mutations sets, except for [Y449S, N501Y] in Figure 2 have positive BFE changes, following the natural selection. We anticipate that although co-mutation sets [V401L, L452R, T478K], [L452R, T478K, N501Y], [A411S, L452R, T478K], and [L452R, T478K, E484K, N501Y] have relatively low frequencies at this point, they may become dangerous variants soon due to their large BFE changes and antibody disruption counts.

**Figure 3:**
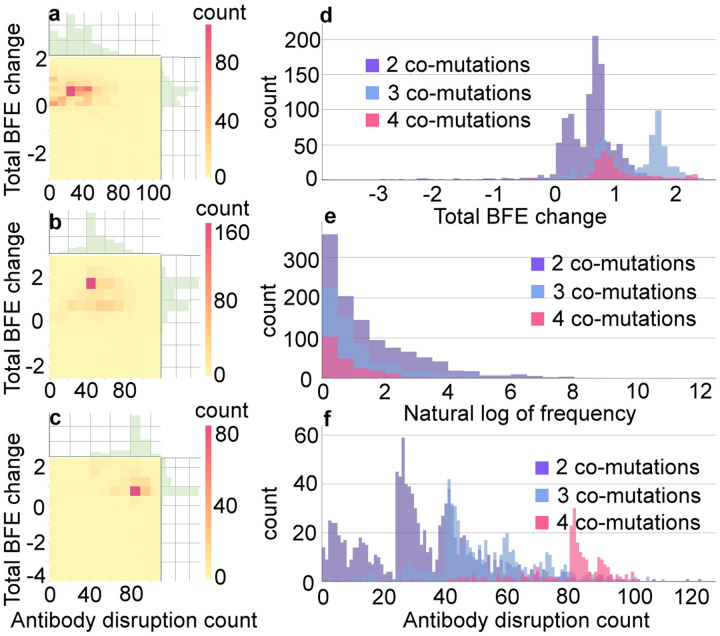
**a** 2D histograms of antibody disruption count and total BFE changes for RBD 2 co-mutations. **b** 2D histograms of antibody disruption count and total BFE changes for RBD 3 co-mutations. **c** 2D histograms of antibody disruption count and total BFE changes for RBD 4 co-mutations. **d** The histograms of total BFE changes for RBD co-mutations. **e** The histograms of the natural log of frequency for RBD co-mutations. **f** The histograms of antibody disruption count for RBD co-mutations. In figures **a**, **b**, and **c**, the color bar represents the number of co-mutations that fall into the restriction of *x*-axis and *y*-axis. The reader is referred to the web version of these plots in the Supporting Information S2.2.2 and S2.2.3.

It is important to understand the general trend of SARS-CoV-2 evolution. To this end, we carry out the statistical analysis of RBD co-mutations. Among 1,489,884 SARS-CoV-2 genome isolates, a total of 1,113 distinctive 2 co-mutations, 612 distinctive 3 co-mutations, and 217 distinctive 4 co-mutations are found. Figures 3 a, b, and c illustrate the 2D histograms of 2, 3, and 4 co-mutations, respectively. The *x*-axis is the number of antibody disruption counts, and the *y*-axis shows the total BFE change. Figure 3 a shows that there are 82 RBD 2 co-mutations that have BFE changes in the range of [0.600, 0.799] kcal/mol and will disruptive 40 to 49 antibodies. According to Figure 3 b, there are 170 unique 3 co-mutations that have large BFE changes of S protein and ACE2 in the range of [1.500, 1.999] kcal/mol. In Figure 3 c, it is seen that almost all of the 4 co-mutations on RBD have the BFE changes greater than 0.5 kcal/mol and weaken the binding of S protein with at least 60 antibodies. Figures 3 d, e, and f are the histograms of total BFE changes, natural log of frequencies, and antibody disruption counts for RBD 2, 3, and 4 co-mutations. It can be found that most of the 2, 3, and 4 RBD co-mutations have positive total BFE changes, and the larger number of RBD co-mutations is, the higher number of antibody disruption count will be. In summary, co-mutations with a larger number of antibody disruptive counts and high BFE changes will grow faster. We anticipate that when most of the population is vaccinated, vaccine-resistant mutations will become a more viable mechanism for viral evolution.

### 2.3 Emerging breakthrough variants in COVID-19 devastated countries

**Figure 4:**
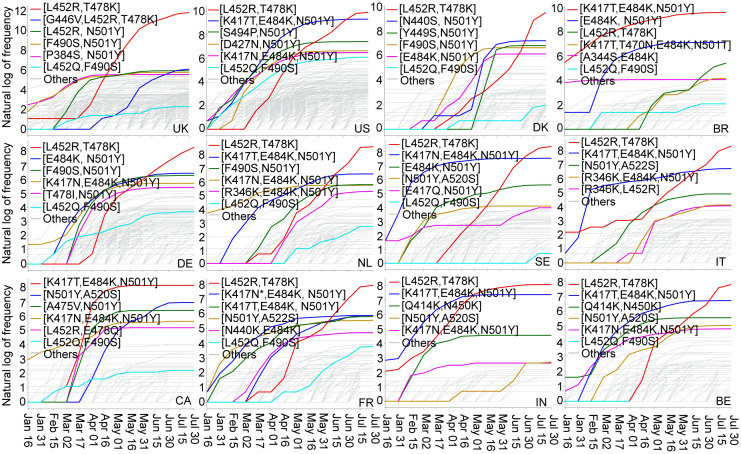
Illustration of the time evolution of 2, 3, and 4 co-mutations on the S protein RBD of SARS-CoV-2 from January 01, 2021, to July 31, 2021, in 12 COVID-19 devastated countries: the United Kingdom (UK), the United States (US), Denmark (DK), Brazil (BR), Germany (DE), Netherlands (NL), Sweden (SE), Italy (IT), Canada (CA), France (FR), India (IN), and Belgium (BE). The *y*-axis represents the natural log frequency of each RBD co-mutation. The top 5 high-frequency co-mutations in each country are marked by red, blue, green, yellow, and pink lines. The cyan line is for the RBD co-mutation [L452Q, F490S] on the Lambda variant, and the other co-mutations are marked by light grey lines. Notably, there are two blues lines in the panel of FR due to the same frequency of [K417N, E484K, N501Y] and [E484K, N501Y]. (Please check the interactive HTML files in the Supporting Information S2.2.1 for a better view of these plots.)

Our analysis of RBD mutations reveals the recent global surge of infections due to RBD co-mutations. However, due to the difference in the rate of vaccination, COVID-19 control and prevention measures, medical infrastructure, population structures, etc., each country may have a different pattern of RBD co-mutations and follow a different trajectory of SARS-CoV-2 transmission and evolution. Therefore, we analyze the RBD 2, 3, and 4 co-mutations in 20 countries that have the high frequency of SARS-CoV-2 genome isolates, including the United Kingdom (UK), the United States (US), Denmark (DK), Brazil (BR), Germany (DE), Netherlands (NL), Sweden (SE), Italy (IT), Canada (CA), France (FR), India (IN), and Belgium (BE), as well as Ireland (IE), Spain (ES), Chile (CL), Portugal (PT), Mexico (MX), Singapore (SG), Turkey (TR), and Finland (FL). Figure 4 shows the time evolution of 2, 3, and 4 co-mutations on the S protein RBD of SARS-CoV-2 from January 01, 2021, to July 31, 2021, in 12 COVID-19 devastated countries. The plots of the other 8 countries can be found in the Supporting Information S3. The top 5 high-frequency co-mutations in each country are marked by red, blue, green, yellow, and pink lines. The cyan line is for the RBD co-mutation set [L452Q, F490S] on the Lambda variant, which is more penetrative to vaccines than the Delta. Light grey lines mark the other co-mutations. The RBD co-mutation set [L452R, T478K] (Delta) with 1.575 kcal/mol BFE change was first found in IN in early January 2021, and the number of this variant increases rapidly around the world in a short period. Later on, in early March 2021, the UK, US, DK, DE, NL, SE, IT, FR, BE reported the appearance of [L452R, T478K] in early March 2021, and eventually [L452R, T478K] became a dominated co-mutation, which is consistent to the finding that Delta variant remains largely susceptible to infection. The co-mutation set [K417T, E484K, N501Y] (Gamma) with BFE change of 0.656 kcal/mol was first found in Brazil in early January 2021, and then it became the most dominated co-mutation in Brazil and Canada, and the second dominated co-mutation in the US, NL, SE, IT, FR, IN, and BE. Notably, co-mutaion set [G446V, L452R, T478K] in the UK with BFE change of 1.733 kcal/mol and 46 antibody disruption counts appears to be a dangerous set of co-mutations that may affect the infectivity and vaccine/antibodies efficacy shortly. Moreover, co-mutation set [N501Y, A520S] has quickly increased IN and BE since April 16, 2021. Considering the BFE change and antibody disruptive count of co-mutation set [N501Y, A520S] is 0.699 and 27, we suggest monitoring this variant in IN and BE. Furthermore, the co-mutation set [K417N, T470N, E484K, N501T] that was first found in BR on April 06, 2020, has a BFE change of 0.625 kcal/mol and antibody disruption count 84, is an emerging vaccine breakthrough co-mutation in Brazil. In addition, co-mutation set [L452Q, F490S] (cyan lines) on Lambda variant was recently drawing much attention due to its potential ability to resist vaccines and enhance the infectivity, which is consistent with our predictions that co-mutation set [L452Q, F490S] has a relatively significant BFE change of S protein and ACE2 (1.421kcal/mol) and would reduce the RBD binding with 59 antibodies. Lambda has already spread out in every country in Figure 4.

## 3 Methodology and validation

**Figure 5:**
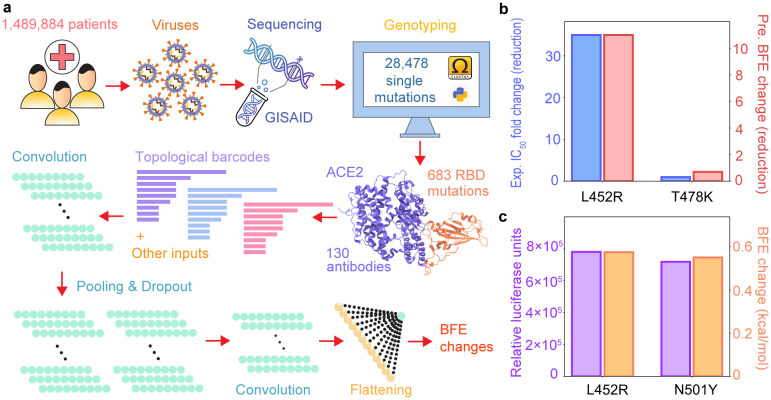
**a** Illustration of genome sequence data pre-processing and BFE change predictions. **b** Comparison of experimental CT-P59 IC_50_ fold change (reduction) and predicted BFE changes induced by mutations L452R and T478K. **c** Comparison of predicted BFE changes and relative luciferase units [25] for pseudovirus infection changes of ACE2 and S protein complex induced by mutations L452R and N501Y.

In this section, the work flow of deep learning-based BFE change predictions of protein-protein interactions induced by mutations for the present SARS-CoV-2 variant analysis and prediction will be firstly introduced, which includes four steps as shown in Figure 5: (1) Data pre-processing; (2) training data preparation; (3) feature generations of protein-protein interaction complexes; (4) prediction of protein-protein interactions by deep neural networks (check Section S5 in Supporting information). Next, the validation of our machine learning-based model will be demonstrated, suggesting consistent and reliable results compared to the experimental deep mutations data.

### 3.1 Data pre-processing and SNP genotyping

The first step is to pre-process the original SARS-CoV-2 sequences data. In this step, a total of 1,489,884 complete SARS-CoV-2 genome sequences with high coverage and exact collection date are downloaded from the GISAID database [34] (https://www.gisaid.org/) as of August 05, 2021. Complete SARS-CoV-2 genome sequences are available from the GISAID database [34]. Next, the 1,489,884 complete SARS-CoV-2 genome sequences were rearranged according to the reference genome downloaded from the GenBank (NC 045512.2) [35], and multiple sequence alignment (MSA) is applied by using Cluster Omega with default parameters. Then, single nucleotide polymorphism (SNP) genotyping is applied to measure the genetic variations between different isolates of SARS-CoV-2 by analyzing the rearranged sequences [36, 37], which is be of paramount importance for tracking the genotype changes during the pandemic. The SNP genotyping captures all of the differences between patients’ sequences and the reference genome, which decodes a total of 28,478 unique single mutations from 1,489,884 complete SARS-CoV-2 genome sequences. Among them, 4,653 non-degenerate mutations on S protein and 683 non-degenerate mutations on the S protein RBD (S protein residues from 329 to 530) are detected. In this work, the co-mutation analysis is more crucial than the unique single mutation analysis. Therefore, for each SARS-CoV-2 isolate, we extract the all of the mutations on S protein RBD, which is called a RBD co-mutation for a specific isolates. By doing this, a total of 1,139,244 RBD co-mutations are captured. Notably, the SARS-CoV-2 unique single mutations in the world is available at Mutation Tracker. The analysis of RBD mutations is available at Mutation Analyzer.

### 3.2 Methods for BFE change predictions

In this section, the process of the machine learning-based BFE change predictions is introduced. Once the data pre-processing and SNP genotyping is carried out, we will firstly proceed with the training data preparation process, which plays a key role in reliability and accuracy. A library of 130 antibodies and RBD complexes as well as an ACE2-RBD complex are obtained from Protein Data Bank (PDB). RBD mutation-induced BFE changes of these complexes are evaluated by the following machine learning model. According to the emergency and the rapid change of RNA virus, it is rare to have massive experimental BFE change data of SARS-CoV-2, while, on the other hand, next-generation sequencing data is relatively easy to collect. In the training process, the dataset of BFE changes induced by mutations of the SKEMPI 2.0 dataset [38] is used as the basic training set, while next-generation sequencing datasets are added as assistant training sets. The SKEMPI 2.0 contains 7,085 single- and multi-point mutations and 4,169 elements of that in 319 different protein complexes used for the machine learning model training. The mutational scanning data consists of experimental data of the binding of ACE2 and RBD induced mutations on ACE2 [39] and RBD [40, 41], and the binding of CTC-445.2 and RBD with mutations on both protein [41].

Next, the feature generations of protein-protein interaction complexes is performed. The element-specific algebraic topological analysis on complex structures is implemented to generate topological bar codes [30, 42–44]. In addition, biochemistry and biophysics features such as Coulomb interactions, surface areas, electrostatics, et al., are combined with topological features [20]. The detailed information about the topology-based models will be demonstrated in subsection 3.3. Lastly, deep neural networks for SARS-CoV-2 are constructed for the BFE change prediction of protein-protein interactions [30]. The detailed descriptions of dataset and machine learning model are found in the literature [19, 30, 45] and are available at TopNetmAb.

### 3.3 Feature generation for machine learning model

Among all features generated for machine learning prediction, the application of topology theory makes the model to a whole new level. Those summarized as other inputs are called as auxiliary features and are described in Section S4 of the Supporting Information. In this section, a brief introduction about the theory of topology will be discussed. Algebraic topology [42,43] has achieved tremendous success in many fields including biochemical and biophysical properties [44]. Special treatment should be implemented for biology applications to describe element types and amino acids in poly-peptide mathematically, which element-specific and site-specific persistent homology [19, 32]. To construct the algebraic topological features on protein-protein interaction model, a series of element subsets for complex structures should be defined, which considers atoms from the mutation sites, atoms in the neighborhood of the mutation site within a certain distance, atoms from antibody binding site, atoms from antigen binding site, and atoms in the system that belong to type of {C, N, O}, $A_{\text{ele}}(\text{E})$. Under the element/site-specific construction, simplicial complexes is constructed on point clouds formed by atoms. For example, a set of independent *k*+1 points is from one element/site-specific set *U* = {*u*_0_*, u*_1_, …, *u*_*k*_}. The *k*-simplex *σ* is a convex hull of *k*+1 independent points *U*, which is a convex combination of independent points. For example, a 0-simplex is a point and a 1-simplex is an edge. Thus, a *m*-face of the *k*-simplex with *m*+1 vertices forms a convex hull in a lower dimension *m* < *k* and is a subset of the *k*+1 vertices of a *k*-simplex, so that a sum of all its (*k*−1)–faces is the boundary of a *k*–simplex *σ* as

(1) $$\partial_{k}\sigma =\sum_{i=1}^{k}{\left(-1\right)}^{i}\langle u_{0},\ldots ,{\hat{u}}_{i},\ldots ,u_{k}\rangle ,$$

where $\langle u_{0},\ldots ,{\hat{u}}_{i},\ldots ,u_{k}\rangle$ consists of all vertices of *σ* excluding *u*_*i*_. The collection of finitely many simplices is a simplicial complex. In the model, the Vietoris-Rips (VR) complex (if and only if $B\left(u_{i_{j}},\;r\right)\cap B\left(u_{i_{j^{\prime }}},\;r\right)\neq \emptyset$ for *j, j*′ ∈ [0*, k*]) is for dimension 0 topology, and alpha complex (if and only if $\cap_{u_{i_{j}}\in \sigma }B\left(u_{i_{j}},\;r\right)\neq \emptyset$) is for point cloud of dimensions 1 and 2 topology [44].

The *k*-chain *c*_*k*_ of a simplicial complex *K* is a formal sum of the *k*-simplices in *K*, which is *c*_*k*_ = ∑*α*_*i*_*σ*_*i*_, where *α*_*i*_ is coefficients and is chosen to be ${\mathbb{Z}}_{2}$. Thus, the boundary operator on a *k*-chain *c*_*k*_ is

(2) $$\partial_{k}c_{k}=\sum \alpha_{i}\partial_{k}\sigma_{i},$$

such that *∂*_*k*_ : *C*_*k*_ → *C*_*k*−1_ and follows from that boundaries are boundaryless *∂*_*k*−1_*∂*_*k*_ = ∅. A chain complex is

(3) $$\cdots \overset{\partial_{i+1}}{\to }C_{i}(K)\overset{\partial_{i}}{\to }C_{i-1}(K)\overset{\partial_{i-1}}{\to }\cdots \overset{\partial_{2}}{\to }C_{1}(K)\overset{\partial_{1}}{\to }C_{0}(K)\overset{\partial_{0}}{\to }0,$$

as a sequence of complexes by boundary maps. Therefore, the Betti numbers are given as the ranks of *k*th homology group *H*_*k*_ as *β*_*k*_ = rank(*H*_*k*_), where *H*_*k*_ = *Z*_*k*_*/B*_*k*_, *k*-cycle group *Z*_*k*_ and the *k*-boundary group *B*_*k*_. The Betti numbers are the key for topological features, where *β*_0_ gives the number of connected components, such as number of atoms, *β*_1_ is the number of cycles in the complex structure, and *β*_2_ illustrates the number of cavities. This presents abstract properties of the 3D structure.

Finally, only one simplicial complex couldn’t give the whole picture of the protein-protein interaction structure. A filtration of a topology space is needed to extract more properties. A filtration is a nested sequence such that

(4) $$\emptyset =K_{0}\subseteq K_{1}\subseteq \cdots \subseteq K_{m}=K.$$

Each element of the sequence could generate the Betti numbers {*β*_0_*, β*_1_*, β*_2_} and consequentially, a series of Betti numbers in three dimensions is constructed and applied to be the topological fingerprints in Figure 5a.

### 3.4 Validation

The validation of our machine learning predictions for mutation-induced BFE changes compared to experimental data has been demonstrated in recently published papers [20, 30]. Firstly, we showed high correlations of experimental deep mutational enrichment data and predictions for the binding complex of SARS-CoV-2 S protein RBD and protein CTC-445.2 [20] and the binding complex of SARS-CoV-2 RBD and ACE2 [30]. In comparison with experimental data on antibody therapies in clinical trials of emerging mutations, our predictions achieve a Pearson correlation at 0.80 [30]. Considering the BFE changes induced by RBD mutations for ACE2 and RBD complex, predictions on mutations L452R and N501Y have a highly similar trend with experimental data [30]. Meanwhile, as we presented in [18], high-frequency mutations are all having positive BFE changes. Moreover, for multi-mutation tests, our BFE change predictions have the same pattern with experimental data of the impact of SARS-CoV-2 variants on major antibody therapeutic candidates, where the BFE changes are accumulative for co-mutations [30].

Recent studies on potency of mAb CT-P59 in vitro and in vivo against Delta variants [46] show that the neutralization of CT-P59 is reduced by L452R (13.22 ng/mL) and is retained against T478K (0.213 ng/mL). In our predictions [30], L452R induces a negative BFE change (−2.39 kcal/mol), and T478K produces a positive BFE change (0.36 kcal/mol). In Figure 5b, the fold changes for experimental and predicted values are presented. Additional, in Figure 5c, a comparison of the experimental pseudovirus infection changes and predicted BFE change of ACE2 and S protein complex induced by mutations L452R and N501Y, where the experimental data is obtained in a reference to D614G and reported in relative luciferase units [25]. It indicates that the binding of RBD and ACE2 dominates the infectivity of SARS-CoV-2. More details can be found in Section S6 of Supporting information.

## Figures and Tables

**Table 1:** Top 25 most observed S protein RBD mutations. Here, BFE change refers to the BFE change for the S protein and human ACE2 complex induced by a single-site S protein RBD mutation. A positive mutation-induced BFE change strengthens the binding between S protein and ACE2, which results in more infectious variants. Counts of antibody disruption represent the number of antibody and S protein complexes disrupted by a specific RBD mutation. Here, an antibody and S protein complex is to be disrupted if its binding affinity is reduced by more than 0.3 kcal/mol [18]. In addition, we calculate the antibody disruption ratio (%), which is the ratio of the number of disrupted antibody and S protein complexes over 130 known complexes. Ranks are computed from 683 observed RBD mutations.

Mutation	Worldwide		BFE change		Antibody disruption		
	Count	Rank	Change	Rank	Count	Ratio	Rank
N501Y	744354	1	0.5499	30	24	18.46	160
L452R	259345	2	0.5752	28	39	30.0	98
T478K	239619	3	0.9994	2	2	1.54	557
E484K	84167	4	0.0946	272	38	29.23	104
K417T	37748	5	0.0116	433	37	28.46	107
S477N	32673	6	0.0180	422	0	0.0	650
N439K	16154	7	0.1792	159	11	8.46	272
K417N	8399	8	0.1661	176	53	40.77	61
F490S	5617	9	0.4406	52	51	39.23	67
S494P	5119	10	0.0902	282	62	47.69	46
N440K	3379	11	0.6161	22	0	0.0	645
E484Q	3229	12	0.0057	442	30	23.08	130
L452Q	2858	13	0.9802	3	27	20.77	144
A520S	2727	14	0.1495	199	3	2.31	497
N501T	2054	15	0.4514	48	17	13.08	202
R357K	1973	16	0.1393	208	5	3.85	388
A522S	1959	17	0.1283	221	2	1.54	543
R346K	1686	18	0.1234	229	6	4.62	380
V367F	1395	19	0.1764	161	0	0.0	637
N440S	1361	20	0.1499	197	2	1.54	542
P384L	1155	21	0.2681	105	18	13.85	199
Y449S	1146	22	−0.8112	632	85	65.38	16
D427N	1106	23	−0.1133	558	1	0.77	589
R346S	1037	24	0.0374	386	20	15.38	182
A475V	891	25	0.3069	94	10	7.69	289

## Data Availability

The SARS-CoV-2 SNP data in the world is available at Mutation Tracker. The most observed SARS-CoV-2 RBD mutations are available at Mutaton Analyzer. The information of 130 antibodies with their corresponding PDB IDs can be found in the Supplementary Data. The SARS-CoV-2 S protein RBD SNP and non-degenerate co-mutations data can be found in Section S2.1.4 of the Supporting Information. The TopNetTree model is available at TopNetmAb.

## Footnotes

1 The average BFE changes of 1149 RBD mutations for the RBD-ACE2 complex is −0.28kcal/mol. Randomly, each RBD mutation has a 50% chance to assume a BFE change above or below −0.28kcal/mol, which leads to 2^100^ =1.276506 × 10^30^ possible states for 100 mutations.

## References

[1] Hoffmann Markus, Kleine-Weber Hannah, Schroeder Simon, Krüger Nadine, Herrler Tanja, Erichsen Sandra, Schiergens Tobias S, Herrler Georg, Wu Nai-Huei, Nitsche Andreas, SARS-CoV-2 cell entry depends on ACE2 and TMPRSS2 and is blocked by a clinically proven protease inhibitor. Cell, 181(2):271–280, 2020. 32142651 10.1016/j.cell.2020.02.052 PMC7102627

[2] Chen Jiahui, Gao Kaifu, Wang Rui, Nguyen Duc Duy, and Wei Guo-Wei. Review of COVID-19 antibody therapies. Annual Review of Biophysics, 50:1–30, 2020. 10.1146/annurev-biophys-062920-063711 PMC8155790 33064571

[3] Chen Peter, Nirula Ajay, Heller Barry, Gottlieb Robert L, Boscia Joseph, Morris Jason, Huhn Gregory, Cardona Jose, Mocherla Bharat, Stosor Valentina, SARS-CoV-2 neutralizing antibody LY-CoV555 in outpatients with COVID-19. New England Journal of Medicine, 384(3):229–237, 2021. 10.1056/NEJMoa2029849 PMC7646625 33113295

[4] Li Wendong, Shi Zhengli, Yu Meng, Ren Wuze, Smith Craig, Epstein Jonathan H, Wang Hanzhong, Crameri Gary, Hu Zhihong, Zhang Huajun, Bats are natural reservoirs of SARS-like coronaviruses. Science, 310(5748):676–679, 2005. 16195424 10.1126/science.1118391

[5] Qu Xiu-Xia, Hao Pei, Song Xi-Jun, Jiang Si-Ming, Liu Yan-Xia, Wang Pei-Gang, Rao Xi, Song Huai-Dong, Wang Sheng-Yue, Zuo Yu, Identification of two critical amino acid residues of the severe acute respiratory syndrome coronavirus spike protein for its variation in zoonotic tropism transition via a double substitution strategy. Journal of Biological Chemistry, 280(33):29588–29595, 2005. 10.1074/jbc.M500662200 PMC8740630 15980414

[6] Song Huai-Dong, Tu Chang-Chun, Zhang Guo-Wei, Wang Sheng-Yue, Zheng Kui, Lei Lian-Cheng, Chen Qiu-Xia, Gao Yu-Wei, Zhou Hui-Qiong, Xiang Hua, Cross-host evolution of severe acute respiratory syndrome coronavirus in palm civet and human. Proceedings of the National Academy of Sciences, 102(7):2430–2435, 2005. 10.1073/pnas.0409608102 PMC548959 15695582

[7] Walls Alexandra C, Park Young-Jun, Tortorici M Alejandra, Wall Abigail, McGuire Andrew T, and Veesler David. Structure, function, and antigenicity of the SARS-CoV-2 spike glycoprotein. Cell, 2020. 10.1016/j.cell.2020.02.058 PMC7102599 32155444

[8] Wang Chunyan, Li Wentao, Drabek Dubravka, Okba Nisreen MA, van Haperen Rien, Osterhaus Albert DME, van Kuppeveld Frank JM, Haagmans Bart L, Grosveld Frank, and Bosch Berend-Jan. A human monoclonal antibody blocking SARS-CoV-2 infection. Nature communications, 11(1):1–6, 2020. 10.1038/s41467-020-16256-y PMC7198537 32366817

[9] Yu Fei, Xiang Rong, Deng Xiaoqian, Wang Lili, Yu Zhengsen, Tian Shijun, Liang Ruiying, Li Yanbai, Ying Tianlei, and Jiang Shibo. Receptor-binding domain-specific human neutralizing monoclonal antibodies against SARS-CoV and SARS-CoV-2. Signal Transduction and Targeted Therapy, 5(1):1–12, 2020. 32963228 10.1038/s41392-020-00318-0 PMC7506210

[10] Li Cheng, Tian Xiaolong, Jia Xiaodong, Wan Jinkai, Lu Lu, Jiang Shibo, Lan Fei, Lu Yinying, Wu Yanling, and Ying Tianlei. The impact of receptor-binding domain natural mutations on antibody recognition of SARS-CoV-2. Signal Transduction and Targeted Therapy, 6(1):1–3, 2021. 33758169 10.1038/s41392-021-00536-0 PMC7985591

[11] Sanjuán Rafael and Domingo-Calap Pilar. Mechanisms of viral mutation. Cellular and Molecular Life Sciences, 73(23):4433–4448, 2016. 27392606 10.1007/s00018-016-2299-6 PMC5075021

[12] Grubaugh Nathan D, Hanage William P, and Rasmussen Angela L. Making sense of mutation: what D614G means for the COVID-19 pandemic remains unclear. Cell, 182(4):794–795, 2020. 32697970 10.1016/j.cell.2020.06.040 PMC7332445

[13] Kucukkal Tugba G, Petukh Marharyta, Li Lin, and Alexov Emil. Structural and physico-chemical effects of disease and non-disease nsSNPs on proteins. Current Opinion in Structural Biology, 32:18–24, 2015. 25658850 10.1016/j.sbi.2015.01.003 PMC4511717

[14] Yue Peng, Li Zhaolong, and Moult John. Loss of protein structure stability as a major causative factor in monogenic disease. Journal of molecular biology, 353(2):459–473, 2005. 16169011 10.1016/j.jmb.2005.08.020

[15] Wang Rui, Hozumi Yuta, Zheng Yong-Hui, Yin Changchuan, and Wei Guo-Wei. Host immune response driving SARS-CoV-2 evolution. Viruses, 12(10):1095, 2020. 10.3390/v12101095 PMC7599751 32992592

[16] Sevajol Marion, Subissi Lorenzo, Decroly Etienne, Canard Bruno, and Imbert Isabelle. Insights into RNA synthesis, capping, and proofreading mechanisms of SARS-coronavirus. Virus Research, 194:90–99, 2014. 25451065 10.1016/j.virusres.2014.10.008 PMC7114481

[17] Ferron François, Subissi Lorenzo, Silveira De Morais Ana Theresa, Le Nhung Thi Tuyet, Sevajol Marion, Gluais Laure, Decroly Etienne, Vonrhein Clemens, Bricogne Gérard, Canard Bruno, Structural and molecular basis of mismatch correction and ribavirin excision from coronavirus RNA. Proceedings of the National Academy of Sciences, 115(2):E162–E171, 2018. 10.1073/pnas.1718806115 PMC5777078 29279395

[18] Wang Rui, Chen Jiahui, Gao Kaifu, and Wei Guo-Wei. Vaccine-escape and fast-growing mutations in the United Kingdom, the United States, Singapore, Spain, India, and other COVID-19-devastated countries. Genomics, 113(4):2158–2170, 2021. 34004284 10.1016/j.ygeno.2021.05.006 PMC8123493

[19] Chen Jiahui, Wang Rui, Wang Menglun, and Wei Guo-Wei. Mutations strengthened SARS-CoV-2 infectivity. Journal of molecular biology, 432(19):5212–5226, 2020. 32710986 10.1016/j.jmb.2020.07.009 PMC7375973

[20] Chen Jiahui, Gao Kaifu, Wang Rui, and Wei Guo-Wei. Prediction and mitigation of mutation threats to COVID-19 vaccines and antibody therapies. Chemical Science, 12(20):6929–6948, 2021. 34123321 10.1039/d1sc01203g PMC8153213

[21] Davies Nicholas G, Abbott Sam, Barnard Rosanna C, Jarvis Christopher I, Kucharski Adam J, Munday James D, Pearson Carl AB, Russell Timothy W, Tully Damien C, Washburne Alex D, Estimated transmissibility and impact of SARS-CoV-2 lineage B. 1.1. 7 in England. Science, 372(6538), 2021. 10.1126/science.abg3055 PMC8128288 33658326

[22] Wang Pengfei, Casner Ryan G, Nair Manoj S, Wang Maple, Yu Jian, Cerutti Gabriele, Liu Lihong, Kwong Peter D, Huang Yaoxing, Shapiro Lawrence, Increased resistance of SARS-CoV-2 variant P. 1 to antibody neutralization. Cell host & microbe, 29(5):747–751, 2021. 33887205 10.1016/j.chom.2021.04.007 PMC8053237

[23] Emary Katherine RW, Golubchik Tanya, Aley Parvinder K, Ariani Cristina V, Angus Brian, Bibi Sagida, Blane Beth, Bonsall David, Cicconi Paola, Charlton Sue, Efficacy of ChAdOx1 nCoV-19 (AZD1222) vaccine against SARS-CoV-2 variant of concern 202012/01 (B. 1.1. 7): an exploratory analysis of a randomised controlled trial. The Lancet, 397(10282):1351–1362, 2021. 10.1016/S0140-6736(21)00628-0 PMC8009612 33798499

[24] Madhi Shabir A, Baillie Vicky, Cutland Clare L, Voysey Merryn, Koen Anthonet L, Fairlie Lee, Padayachee Sherman D, Dheda Keertan, Barnabas Shaun L, Bhorat Qasim E, Efficacy of the ChAdOx1 nCoV-19 COVID-19 vaccine against the B. 1.351 variant. New England Journal of Medicine, 384(20):1885–1898, 2021. 10.1056/NEJMoa2102214 PMC7993410 33725432

[25] Deng Xianding, Garcia-Knight Miguel A, Khalid Mir M, Servellita Venice, Wang Candace, Morris Mary Kate, Sotomayor-González Alicia, Glasner Dustin R, Reyes Kevin R, Gliwa Amelia S, Transmission, infectivity, and antibody neutralization of an emerging SARS-CoV-2 variant in California carrying a L452R spike protein mutation. MedRxiv, 2021. 10.1016/j.cell.2021.04.025 PMC8057738 33991487

[26] Jangra Sonia, Ye Chengjin, Rathnasinghe Raveen, Stadlbauer Daniel, Alshammary Hala, Amoako Angela A, Awawda Mahmoud H, Beach Katherine F, Bermúdez-González Maria C, Chernet Rachel L, SARS-CoV-2 spike E484K mutation reduces antibody neutralisation. The Lancet Microbe, 2021. 10.1016/S2666-5247(21)00068-9 PMC8026167 33846703

[27] Annavajhala Medini K, Mohri Hiroshi, Zucker Jason E, Sheng Zizhang, Wang Pengfei, Gomez-Simmonds Angela, Ho David D, and Uhlemann Anne-Catrin. A novel SARS-CoV-2 variant of concern, B. 1.526, identified in New York. medRxiv, 2021.

[28] Greaney Allison J, Loes Andrea N, Crawford Katharine HD, Starr Tyler N, Malone Keara D, Chu Helen Y, and Bloom Jesse D. Comprehensive mapping of mutations in the SARS-CoV-2 receptor-binding domain that affect recognition by polyclonal human plasma antibodies. Cell host & microbe, 29(3):463–476, 2021. 33592168 10.1016/j.chom.2021.02.003 PMC7869748

[29] Kimura Izumi, Kosugi Yusuke, Wu Jiaqi, Yamasoba Daichi, Butlertanaka Erika P, Tanaka Yuri L, Liu Yafei, Shirakawa Kotaro, Kazuma Yasuhiro, Nomura Ryosuke, SARS-CoV-2 Lambda variant exhibits higher infectivity and immune resistance. bioRxiv, 2021. 10.1016/j.celrep.2021.110218 PMC8683271 34968415

[30] Chen Jiahui, Gao Kaifu, Wang Rui, and Wei Guo-Wei. Revealing the threat of emerging SARS-CoV-2 mutations to antibody therapies. Journal of Molecular Biology, 433(7744), 2021. 10.1016/j.jmb.2021.167155 PMC8277955 34273397

[31] Nguyen Duc Duy, Cang Zixuan, Wu Kedi, Wang Menglun, Cao Yin, and Wei Guo-Wei. Mathematical deep learning for pose and binding affinity prediction and ranking in D3R Grand Challenges. Journal of Computer-aided Molecular Design, 33(1):71–82, 2019. 30116918 10.1007/s10822-018-0146-6 PMC7163798

[32] Wang Menglun, Cang Zixuan, and Wei Guo-Wei. A topology-based network tree for the prediction of protein–protein binding affinity changes following mutation. Nature Machine Intelligence, 2(2):116–123, 2020. 10.1038/s42256-020-0149-6 PMC7223817 34170981

[33] Cherian Sarah, Potdar Varsha, Jadhav Santosh, Yadav Pragya, Gupta Nivedita, Das Mousumi, Rakshit Partha, Singh Sujeet, Abraham Priya, Panda Samiran, SARS-CoV-2 Spike Mutations, L452R, T478K, E484Q and P681R, in the Second Wave of COVID-19 in Maharashtra, India. Microorganisms, 9(7):1542, 2021. 34361977 10.3390/microorganisms9071542 PMC8307577

[34] Shu Yuelong and McCauley John. GISAID: Global initiative on sharing all influenza data–from vision to reality. Eurosurveillance, 22(13):30494, 2017. 28382917 10.2807/1560-7917.ES.2017.22.13.30494 PMC5388101

[35] Wu Fan, Zhao Su, Yu Bin, Chen Yan-Mei, Wang Wen, Song Zhi-Gang, Hu Yi, Tao Zhao-Wu, Tian Jun-Hua, Pei Yuan-Yuan, A new coronavirus associated with human respiratory disease in China. Nature, 579(7798):265–269, 2020. 32015508 10.1038/s41586-020-2008-3 PMC7094943

[36] Yin Changchuan. Genotyping coronavirus SARS-CoV-2: methods and implications. Genomics, 112(5):3588–3596, 2020. 32353474 10.1016/j.ygeno.2020.04.016 PMC7184998

[37] Kim Sobin and Misra Ashish. Snp genotyping: technologies and biomedical applications. Annu. Rev. Biomed. Eng., 9:289–320, 2007. 17391067 10.1146/annurev.bioeng.9.060906.152037

[38] Jankauskaitė Justina, Jiménez-García Brian, Dapkūnas Justas, Fernández-Recio Juan, and Moal Iain H. SKEMPI 2.0: an updated benchmark of changes in protein–protein binding energy, kinetics and thermodynamics upon mutation. Bioinformatics, 35(3):462–469, 2019. 30020414 10.1093/bioinformatics/bty635 PMC6361233

[39] Procko Erik. The sequence of human ace2 is suboptimal for binding the s spike protein of sars coronavirus 2. BioRxiv, 2020.

[40] Starr Tyler N, Greaney Allison J, Hilton Sarah K, Ellis Daniel, Crawford Katharine HD, Dingens Adam S, Navarro Mary Jane, Bowen John E, Tortorici M Alejandra, Walls Alexandra C, Deep mutational scanning of SARS-CoV-2 receptor binding domain reveals constraints on folding and ACE2 binding. Cell, 182(5):1295–1310, 2020. 32841599 10.1016/j.cell.2020.08.012 PMC7418704

[41] Linsky Thomas W, Vergara Renan, Codina Nuria, Nelson Jorgen W, Walker Matthew J, Su Wen, Barnes Christopher O, Hsiang Tien-Ying, Esser-Nobis Katharina, Yu Kevin, De novo design of potent and resilient hACE2 decoys to neutralize SARS-CoV-2. Science, 370(6521):1208–1214, 2020. 33154107 10.1126/science.abe0075 PMC7920261

[42] Carlsson Gunnar. Topology and data. Bulletin of the American Mathematical Society, 46(2):255–308, 2009.

[43] Edelsbrunner Herbert, Letscher David, and Zomorodian Afra. Topological persistence and simplification. In Proceedings 41st annual symposium on foundations of computer science, pages 454–463. IEEE, 2000.

[44] Xia Kelin and Wei Guo-Wei. Persistent homology analysis of protein structure, flexibility, and folding. International journal for numerical methods in biomedical engineering, 30(8):814–844, 2014. 24902720 10.1002/cnm.2655 PMC4131872

[45] Wang Rui, Hozumi Yuta, Yin Changchuan, and Wei Guo-Wei. Mutations on COVID-19 diagnostic targets. Genomics, 112(6):5204–5213, 2020. 32966857 10.1016/j.ygeno.2020.09.028 PMC7502284

[46] Lee Soo-Young, Ryu Dong-Kyun, Noh Hanmi, Kim Jongin, Seo Ji-Min, Kim Cheolmin, van Baalen Carel, Tijsma Aloys SL, Chung Hyo-Young, Lee Min-Ho, Therapeutic efficacy of CT-p59 against P. 1 variant of SARS-CoV-2. bioRxiv, 2021.

